# Prediction of tumor deposits in stage I-III gastric cancer: a clinically applicable nomogram integrating clinicopathology outcomes

**DOI:** 10.3389/fmed.2025.1577569

**Published:** 2025-05-30

**Authors:** Kunjie Wang, Yue Huo, Yuanfang Zhang, Song Guo, Weiguang Yu, Na Xiao, Shenyong Su, Lin An

**Affiliations:** ^1^Department of Medical Oncology, Affiliated Hospital of Hebei University, Hebei Key Laboratory of Cancer Radiotherapy and Chemotherapy, Baoding, Hebei, China; ^2^Department of Emergency Surgery and Orthopaedics, The First Affiliated Hospital, Sun Yat-sen University, Guangzhou, China

**Keywords:** gastric tumor, tumor deposits, risk factors, predictive model, nomogram

## Abstract

**Objective:**

This study seeks to identify clinicopathological risk factors associated with tumor deposits (TD) development in stage I-III gastric cancer patients and to construct a visualized predictive model for clinical application.

**Methods:**

A retrospective cohort of 1,284 gastric cancer patients treated at the Affiliated Hospital of Hebei University (September 2010–September 2022) was analyzed. Patients were stratified into training (*n* = 963) and validation (*n* = 321) cohorts via simple randomization at a 3:1 ratio. Lasso regression analysis was employed to screen variables, followed by multivariate logistic regression to establish an individualized nomogram. Model performance was evaluated using the area under the receiver operating characteristic curve (AUC), calibration plots, and decision curve analysis (DCA).

**Results:**

TD-positive patients (*n* = 224) exhibited significantly reduced overall survival and disease-free survival compared to TD-negative counterparts (*n* = 1,060, *p* < 0.05). Multivariate logistic regression analysis confirmed tumor size (OR = 1.26; 95% CI 1.01–2.21), elevated CEA (OR = 2.04; 95% CI 1.02–3.16), elevated CA199 (OR = 1.007, 95% CI:1.003–1.011), and pN stage (OR = 3.22; 95% CI 2.12–4.34) as independent predictors of TD occurrence (all *p* < 0.05). The nomogram demonstrated robust discriminative capacity, with AUC values of 0.803 (95% CI 0.751–0.894) and 0.864 (95% CI 0.725–0.917) in the training and validation cohorts, respectively. Calibration plots revealed excellent agreement between predicted and observed probabilities. DCA further validated the model’s clinical utility, showing superior net benefits across threshold probabilities of 1–99%.

**Conclusion:**

This TD-specific nomogram, incorporating tumor size, serum biomarkers (CEA/CA199), and pathological staging (pN), provides a clinically applicable tool for preoperative risk stratification and personalized therapeutic decision-making in stage I-III gastric cancer.

## Introduction

Gastric adenocarcinoma remains a formidable global health burden, ranking as the fifth most prevalent malignancy and the fourth leading cause of cancer-related mortality worldwide (1–6). While diagnostic advancements have improved early-stage detection, approximately 30% of patients with clinically localized disease (Stage I-III) experience unexpected failure, highlighting critical gaps in current prognostic stratification systems (7–10). Emerging evidence identifies tumor deposits (TDs) - discrete perigastric neoplastic nodules distinct from lymphatic metastases - as pivotal determinants of adverse oncological outcomes (11–15). These extranodal tumor manifestations demonstrate strong correlations with occult micrometastatic dissemination, vascular invasion patterns, and significantly reduced survival rates across multiple cohorts (16–19).

Contemporary studies (16, 18, 20–22) have established TD presence as an independent prognostic variable in gastric cancer. Despite this clinical significance, current TNM staging paradigms inadequately address TD quantification, creating prognostic ambiguity for approximately 15%–20% of Stage II-III patients (2–4). Furthermore, existing predictive models for TD occurrence exhibit critical limitations, including restricted variable selection (e.g., omitting emerging biomarkers like systemic immune-inflammatory indices) and insufficient validation across diverse populations, particularly in multiethnic cohorts and geographically distinct healthcare settings (16, 19, 21).

This study addresses these clinical and methodological gaps through three principal objectives: First, to identify novel clinicopathological and molecular determinants of TD formation using machine learning-enhanced multivariate regression. Second, to develop and externally validate a TD-specific prognostic nomogram integrating pathological staging and serum biomarker profiles. Third, to establish an open-access digital risk stratification tool enabling real-time TD probability estimation. Building upon the foundational work (23) in gastric cancer risk modeling, our methodology incorporates advanced ensemble learning algorithms and bootstrap validation to optimize discriminatory capacity (target AUC > 0.85), while maintaining clinical interpretability.

By reconciling molecular pathogenesis with clinical decision-making needs, this investigation advances the paradigm of precision prognostication in gastric oncology. The resultant predictive framework not only refines therapeutic stratification but also provides a template for incorporating complex tumor microenvironment features into standardized staging systems.

## Materials and methods

### Study design and ethical compliance

This retrospective cohort study adhered to the ethical principles outlined in the Declaration of Helsinki and received approval from the Institutional Review Board of the Affiliated Hospital of Hebei University (Approval No. 32017). Informed consent was waived in compliance with national regulations governing retrospective analyses of anonymized clinical data. Patient identifiers were systematically redacted during preprocessing to ensure confidentiality.

### Patient selection and exclusion criteria

Consecutive patients with histopathologically confirmed Stage I-III gastric adenocarcinoma (AJCC 8th Edition TNM criteria) treated between September 2012 and September 2022 were screened. Inclusion criteria comprised: (I) Curative-intent surgery: Radical gastrectomy (total/subtotal) with D1/D2 lymphadenectomy; (II) Clinicopathological completeness: Demographic profiles, tumor characteristics (size, differentiation, Lauren classification), preoperative biomarkers (CEA, CA199, albumin), and surgical-pathological parameters (pT stage, lymph node yield ≥ 15, R0 resection); (III) Follow-up adequacy: Minimum 12-month postoperative surveillance or mortality documentation. Exclusions targeted potential confounders: (I) Metastatic disease (Stage IV) or synchronous malignancies; (II) Neoadjuvant therapy recipients (chemotherapy/radiotherapy); (III) Incomplete nodal dissection (<15 lymph nodes examined); (IV) Non-curative resection (R1/R2 status) or incomplete medical records.

### Propensity score matching

To address potential confounding factors, we performed propensity score matching (PSM) using the nearest-neighbor algorithm with a caliper width of 0.1 and a 1:2 matching ratio. Key covariates included tumor size, serum biomarkers (CEA, CA199), pathological staging (pT/pN), and Lauren classification. The balance of covariates before and after matching was assessed using standardized mean differences (SMD < 0.1 indicating good balance). Propensity scores were estimated via logistic regression incorporating all covariates.

### Data abstraction and definitions

Clinicopathological variables—including demographics, tumor dimensions, Lauren classification, lymphovascular/perineural invasion status, and preoperative biomarkers—were extracted from institutional electronic health records. TDs were rigorously defined per AJCC 8th Edition criteria: discrete perigastric neoplastic nodules within lymphatic drainage territories, devoid of residual lymph node architecture, vascular channels, or neural structures (13).

### Surveillance protocol

Standardized follow-up included quarterly clinical evaluations (imaging, tumor marker assays, physical examination) for the first 24 months after treatment, transitioning to semiannual assessments thereafter. Surveillance concluded in September 2022, with censoring at last confirmed contact or mortality.

### Statistical analysis

Data analysis was executed using R software (version 4.4.3). Continuous variables were examined for normality using the Kolmogorov-Smirnov test. Non-normally distributed data were summarized as median (interquartile range) and compared between groups using the Mann-Whitney U test. Categorical variables were expressed as frequencies (percentages) and analyzed with chi-square or Fisher’s exact tests, as appropriate. Survival curves (overall survival[OS] and progression free survival[PFS]) were generated using the Kaplan-Meier method, with between-group survival rate comparisons performed via log-rank testing. For predictive modeling, tumor deposit occurrence in the training cohort served as the outcome variable. Independent predictors were identified through Least Absolute Shrinkage and Selection Operator (LASSO) regression. Subsequently, a multivariate logistic regression model incorporating these predictors was constructed to develop a nomogram. Model validation was performed on the independent validation cohort: discrimination accuracy was quantified using the area under the receiver operating characteristic (ROC) curve (AUC), calibration was assessed via calibration plots, and clinical utility was evaluated through decision curve analysis (DCA). Statistical significance was established at *p* < 0.05.

## Results

### Propensity score matching outcomes

**FIGURE 1:**
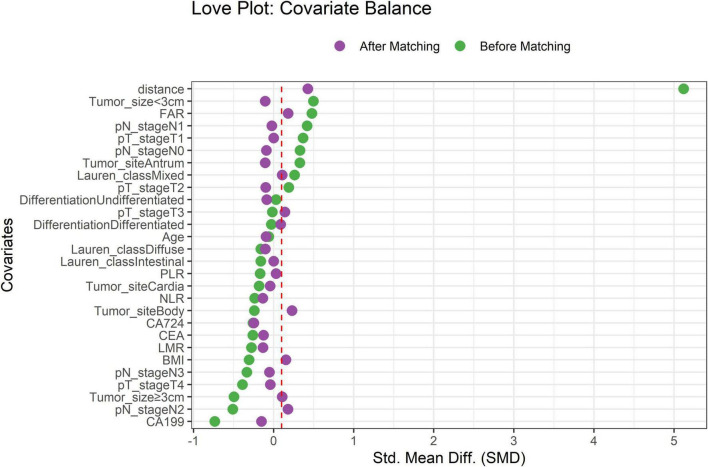
Covariate balance before and after propensity score matching. Standardized mean differences (SMD) for key covariates. Dashed red line indicates the SMD threshold of 0.1.

**FIGURE 2:**
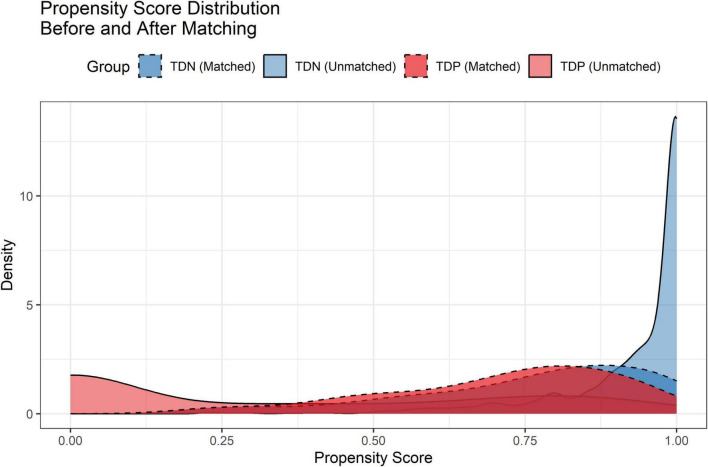
Propensity score distributions before and after matching. Density curves illustrate the overlap between TD-negative (TDN) and TD-positive (TDP) groups post-matching.

After PSM, 224 TD-positive (TDP) and 1,060 TD-negative (TDN) patients were successfully matched. The Love plot (Figure 1) demonstrated significant improvement in covariate balance, with all post-matching SMD values below 0.1. The propensity score distributions (Figure 2) showed substantial overlap between matched groups, confirming reduced selection bias.

### Clinical characteristics and survival outcomes

TDP patients exhibited distinct clinicopathological profiles, including larger tumor dimensions (median size: 4.3 vs. 2.8 cm, *p* < 0.001), elevated serum biomarkers (CEA: 2.6 vs. 2.1 ng/mL; CA199: 17.3 vs. 3.1 U/mL; *p* < 0.001), and higher rates of lymphovascular invasion (58.0% vs. 40.5%) and perineural invasion (38.4% vs. 11.5%) (Table 1).

**TABLE 1:** Baseline data.

Variable	TDP (*n* = 224)	TDN (*n* = 1060)	*p*-value
Age (years)	65 (45–85)	64 (44–86)	0.145^a^
Tumor size/diameter (cm)			0.001^a^
≥3 cm	150	450	
<3 cm	74	610	
BMI	24.3 (22.3–26.4)	23.5 (21.7–25.8)	0.440^a^
CEA (ng/ml)	2.6 (2.3–4.6)	2.1 (1.5–4.3)	0.001^a^
CA724 (u/ml)	2.4 (1.2–3.8)	1.9 (0.6–3.5)	0.001^a^
CA199 (u/ml)	17.3 (4.8–28.4)	13.1 (4.1–32.4)	0.001^a^
FAR	0.7 (0.5–0.9)	0.8 (0.6–0.9)	0.027^a^
PLR	124(110.2–165.4)	117.3(90.8–176.1)	0.016^a^
NLR	1.9 (1.1–2.8)	1.7 (1.2–2.6)	0.304^a^
LMR	7.5 (5.2–8.3)	7.0 (6.3–9.1)	0.038^a^
Tumor site, *n* (%)			0.001^b^
Antrum	95 (42.4)	620 (58.5)	
Body	43 (19.2)	122 (11.5)	
Cardia	86 (38.4)	318 (30.0)	
Lauren’s classification, *n* (%)			0.003^b^
Diffuse type	130 (58.0)	530 (50.0)	
Intestinal type	32 (14.3)	101 (9.5)	
Mixed type	62 (27.7)	429 (40.5)	
Degree of differentiation, *n* (%)			0.682^b^
Differentiated	193 (86.2)	902 (85.0)	
Undifferentiated	31 (13.8)	158 (15.0)	
pT, *n* (%)			0.001^b^
T1	0 (0)	126 (11.9)	
T2	32 (14.3)	234 (22.1)	
T3	64 (28.6)	295 (27.8)	
T4	128 (57.1)	405 (38.2)	
pN, *n* (%)			0.001^b^
N0	34 (15.2)	321 (30.3)	
N1	23 (10.3)	310 (29.2)	
N2	94 (41.9)	224 (21.1)	
N3	73 (32.6)	205 (19.3)	

^a^T-test;

^b^Mann–Whitney U test. TDP, tumor deposit positive; TDN, tumor deposit negative; BMI, body mass index; CEA, carcinoembryonic antigen; CA724, carbohydrate antigen724; CA199, carbohydrate antigen199; FAR, fibrinogen albumin ratio; PLR, platelet lymphocyte ratio; NLR, neutrophil lymphocyte ratio; LMR, lymphocyte monocyte ratio.

### Survival outcomes

**FIGURE 3:**
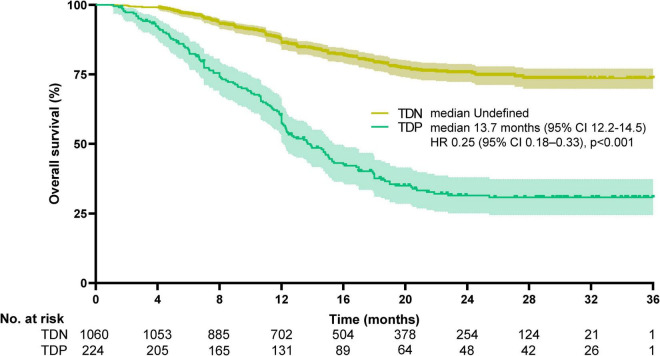
Kaplan-Meier curves for overall survival. The median overall survival was undefined for TDN and 13.7 months (95%CI, 12.2–14.5) for TDP (HR 0.25, 95%CI, 0.18–0.33, *p* < 0.001). Median OS in the TDN group is undefined as >50% of patients were alive at the final follow-up. Landmark survival rates are provided for clinical interpretation.

**FIGURE 4:**
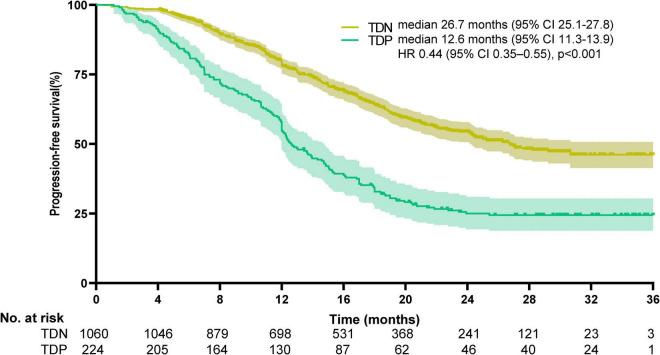
Kaplan-Meier curves for progression-free survival. The median progression-free survival was 26.7 months (95% CI, 25.1–27.8) for TDN and 12.6 months (95%CI, 11.3–13.9) for TDP (HR 0.44, 95%CI, 0.35–0.55; *p* < 0.001).

Survival disparities were pronounced: TDP patients demonstrated a median OS of 13.7 months (95% CI: 12.2–14.5), whereas the median OS for TDN patients remained undefined due to >50% of patients surviving beyond the study period. To better characterize long-term outcomes, we report landmark survival rates: the 1-, 3-, and 5-year OS rates for TDN patients were 94.2, 73.6, and 58.9%, respectively, compared to 60.1, 30.8, and 18.3% in the TDP group (log-rank *p* < 0.001). The HR for the TDN versus TDP groups was reported at 0.25, with a 95%CI between 0.18 and 0.33, indicating that TDN patients had a 75% lower risk of death compared to TDP patients (log-rank *p* < 0.001). The Kaplan-Meier survival curve illustrates that the TDN group maintained a higher OS percentage compared to the TDP group (Figure 3). Similarly, PFS was significantly shorter in TDP patients (median PFS: 12.6 vs. 26.7 months). The HR for the TDN group relative to the TDP group was calculated at 0.44, accompanied by a 95% CI from 0.35 to 0.55 (log-rank *p* < 0.001). This data highlights that patients in the TDN group have nearly halved the risk of disease progression compared to those in the TDP group. The survival curve further illustrates the sustained advantage for the TDN group over time (Figure 4), confirming the adverse prognostic significance of TD status in gastric cancer progression. The 3-year OS rate further emphasized this divergence (TDN: 73.6% vs. TDP: 30.8%).

### Risk factor identification for TD formation

**FIGURE 5:**
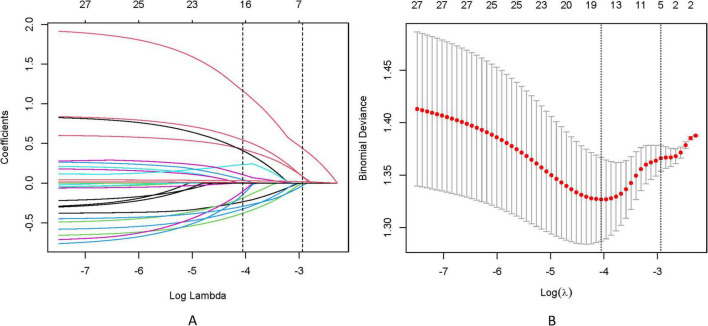
**(A)** LASSO coefficient trajectories depict the shrinkage of 27 predictor variables for TD formation as the penalty parameter (λ) increases. **(B)** Cross-validation results identify the optimal λ (λmin) and a simplified yet robust model (λ1SE), with annotations indicating the number of retained variables.

To address multicollinearity among 27 candidate clinical-pathological variables (Supplementary Table 1), we employed LASSO regression with 10-fold cross-validation, which applies an L1 penalty to shrink coefficients of non-informative variables while retaining predictors with the strongest associations with TD formation (Figures 5A, B). We systematically analyzed the univariate outcomes and reconstructed the dataset using variables with *p*-values less than 0.05. The univariate analysis results are systematically delineated in Table 2, providing an initial insight into the data set. Subsequently, six variables (tumor size, CEA, CA199, pT, pN, and CA724) were retained at the optimal λ threshold, collectively explaining 85.3% of the deviance in TD risk (Table 3). Although CA724 exhibited moderate predictive value in LASSO regression (% deviance = 25.3), it was excluded from the final multivariable model due to its overlapping biological pathways with other biomarkers. This analytical framework prioritized variables based on both statistical significance (coefficients > 0.1) and clinical relevance to tumor biology, thereby adjusting for potential confounding factors and enhancing validity. Multivariable logistic regression analysis, as detailed in Table 4, elucidated that four specific variables as independent predictors of TD occurrence: tumor size (OR = 1.26; 95% CI 1.01–2.21), elevated CEA (OR = 2.04; 95% CI 1.02–3.16), elevated CA199 (OR = 5.17; 95% CI 3.14–7.38), and pN (OR = 3.22; 95% CI 2.12–4.34).

**TABLE 2:** The results of the univariate analysis.

Characteristics	B	SE	OR	CI	*Z*	*P*
Age (years)	0.065	0.713	0.225	0.13–2.31	1.332	0.523
Tumor diameter ≤ 3 cm	1.816	0.214	5.71	4.35–9.14	9.244	0
Tumor diameter < 3 cm	1.271	0.342	6.36	4.61–10.04	12.126	0
BMI	0.422	0.29	1.402	0.87–2.35	1.744	0.321
CEA	0.332	0.221	0.518	0.36–0.79	0.830	0
CA724	0.441	0.255	1.274	0.75–2.01	1.643	0.847
CA199	0.193	0.102	1.519	1.12–2.37	2.791	0
FAR	−0.146	0.237	1.224	0.92–2.03	−5.611	0.073
PLR	−0.273	0.399	1.370	0.81–2.83	−4.758	0.374
NLR	−0.362	0.448	1.377	0.84–1.74	−0.614	0.339
LMR	−0.146	0.235	1.538	0.71–2.51	−4.325	0.891
Antrum	−0.313	0.241	1.594	0.76–2.23	−3.101	0.800
Body	−0.171	0.104	0.312	0.21–1.23	2.24	0.360
Cardia	0.347	0.179	0.575	0.42–2.10	2.02	0.201
Diffuse type	−0.384	0.121	0.929	0.81–2.31	1.26	0.704
Intestinal type	0.227	0.164	1.204	0.48–2.51	2.82	0.146
Mixed type	−0.264	0.193	0.730	0.31–1.17	1.37	0.147
Differentiated	−0.135	0.076	0.748	0.53–1.64	1.94	0.215
Undifferentiated	−0.435	0.373	0.915	0.78–1.81	2.36	0.670
T1	1.147	0.168	2.557	1.27–3.53	4.05	0.005
T2	1.114	0.214	2.546	1.42–3.78	2.49	0.010
T3	1.002	0.101	1.554	1.22–2.39	3.69	0.032
T4	0.451	0.407	3.485	2.49–4.91	6.50	0.037
N0	1.276	0.142	2.556	1.6–3.13	4.61	0
N1	1.403	0.384	2.405	1.86–3.56	6.70	0
N2	1.641	0.452	2.243	1.73–3.43	4.68	0.003
N3	1.737	0.339	10.598	1.13–2.75	3.77	0.014

BMI, body mass index; CEA, carcinoembryonic antigen; CA724, carbohydrate Antigen724; CA199, carbohydrate antigen199; FAR, fibrinogen albumin ratio; PLR, platelet lymphocyte ratio; NLR, neutrophil lymphocyte ratio; LMR, lymphocyte monocyte ratio.

**TABLE 3:** Coefficients and lambda.1SE value of the LASSO regression.

Variable	Df% dev	Lambda
Tumor size	32.4	0.0117
CEA	9.07	0.02318
CA199	10.91	0.01624
pT	7.25	0.04015
pN	27.53	0.02537
CA724	25.3	0.02724

CEA, carcinoembryonic antigen; CA724, carbohydrate antigen724; CA199, carbohydrate antigen199.

**TABLE 4:** Multivariable logistic regression analysis of clinical predictors of TD formation.

Variable	B	SE	OR	CI	*Z*	*P*
Tumor size	0.753	0.151	1.26	1.01–2.21	7.012	<0.001
CEA	0.281	0.162	2.04	1.02–3.16	4.035	<0.001
pN	1.173	0.137	3.22	2.12–4.34	5.426	<0.001
CA199	1.261	0.141	5.17	3.14–7.38	8.261	<0.001

CEA, carcinoembryonic antigen; CA199, carbohydrate antigen199.

### Development and validation of the predictive nomogram

**FIGURE 6:**
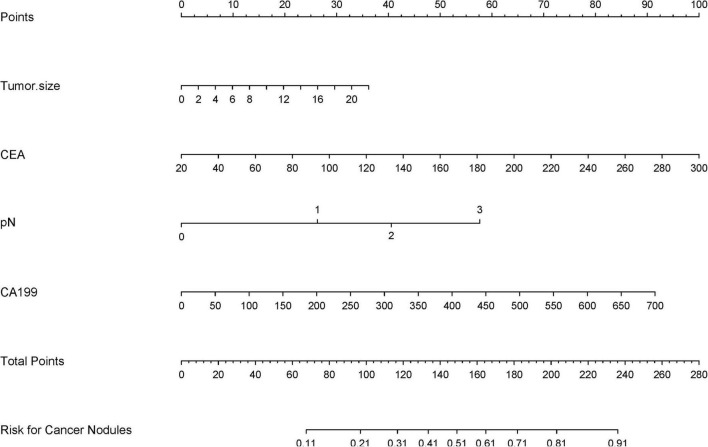
A clinical nomogram, derived from LASSO-selected predictors and multivariable logistic regression, quantifies TD risk by assigning scores to key variables. Total scores map to a probability scale, enabling early TD risk stratification in high-risk populations.

**FIGURE 7:**
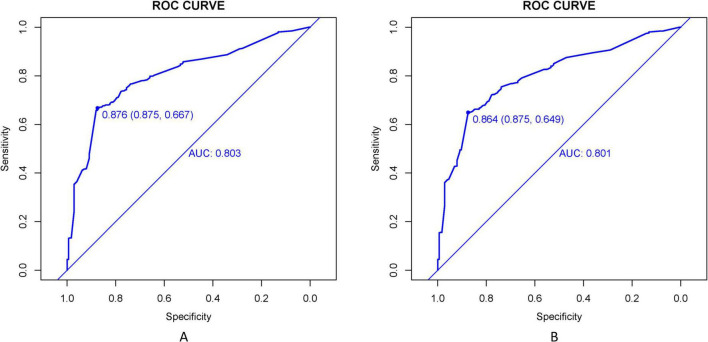
ROC curves evaluate the TD prediction model’s performance in training **(A)** and validation **(B)** cohorts. AUC values reflect the model’s ability to distinguish TD cases across sensitivity-specificity trade-offs at varying thresholds.

The LASSO-derived nomogram integrating these predictors demonstrated robust discriminative capacity (Figure 6). In the training cohort, the model achieved an AUC of 0.803 (95% CI 0.751–0.894) with sensitivity of 87.5% and specificity of 66.7%, while validation cohort performance remained strong (AUC = 0.864; 95% CI: 0.725–0.917; sensitivity = 87.5%, specificity = 64.9%) (Figures 7A, B).

**FIGURE 8:**
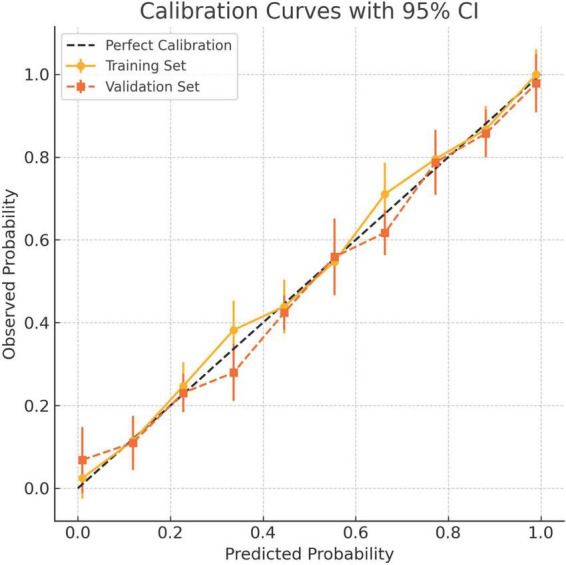
Calibration curves for the 8-factor prediction model in both the training and validation cohorts. The *x*-axis denotes predicted probability of tumor deposits (TD), while the *y*-axis shows the observed frequency in each risk decile. Solid circles indicate the training cohort and squares represent the validation cohort. Vertical bars denote 95% confidence intervals. The dashed diagonal line reflects perfect calibration. The model demonstrates good agreement between predicted and observed risks across both cohorts.

**FIGURE 9:**
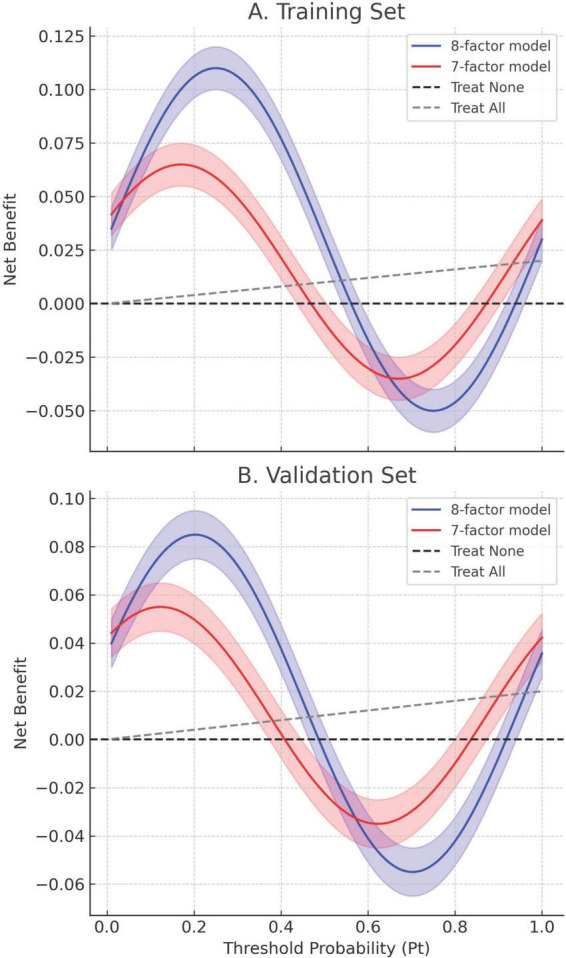
Decision curve analysis (DCA) of the 8-factor and 7-factor models in both the training and validation cohorts. **(A)** Training set. **(B)** Validation set. Net benefit is plotted against threshold probability (Pt), which represents the minimum risk at which intervention would be considered. Solid lines correspond to the estimated net benefit of the 8-factor (blue) and 7-factor (red) models; shaded regions denote 95% confidence intervals derived from bootstrap resampling (1,000 iterations). The dashed black and gray lines represent “Treat None” and “Treat All” strategies, respectively. Across both datasets, the 8-factor model consistently provides greater net benefit over a wide range of Pt values (0.1–0.9), indicating its superior clinical utility for preoperative decision-making.

Calibration accuracy was validated through bootstrap-corrected curves (training R^2^ = 0.252; validation R^2^ = 0.269), with minimal prediction error (Brier score: 0.166 vs. 0.164) (Figure 8). DCA confirmed superior net clinical benefit across threshold probabilities (1%–99%) compared to universal treatment (treat all patients regardless of risk) or no intervention (treat no patients) strategies (Figures 9A, B), underscoring its utility in preoperative risk stratification.

## Discussion

This study establishes tumor diameter, elevated serum CEA/CA199 levels, and advanced pN stage as independent predictors of TD formation in stage I-III gastric cancer. Our machine learning-enhanced nomogram, integrating these clinicopathological variables, demonstrated robust predictive accuracy (training AUC = 0.803; validation AUC = 0.864), addressing a critical unmet need for preoperative risk stratification in gastric oncology. Notably, this performance surpasses widely used tools such as the AJCC 8th Edition TNM staging system (AUC ≈ 0.65–0.70) (12, 14) and recent TD-specific models like Fujikawa et al. (AUC = 0.76) (17), highlighting its clinical superiority.

The dose-dependent relationship between tumor size and TD risk (OR = 1.26 per 1 cm increase) extends prior evidence linking tumor bulk to metastatic dissemination. This finding positions tumor diameter not merely as a categorical marker but as a continuous biological driver of TD pathogenesis, potentially reflecting increased invasive potential in larger lesions (12, 13, 19, 24). Similarly, the prognostic significance of elevated CEA and CA199 aligns with their established roles in epithelial-mesenchymal transition and systemic micrometastasis (2, 3, 25–28). CA199, a sialylated Lewis antigen, further contributes to TD formation through multifaceted mechanisms (29, 30): (1) promoting epithelial-mesenchymal transition via downregulation of E-cadherin and upregulation of vimentin; (2) fostering immune evasion by binding to selectins on immune cells, thereby suppressing cytotoxic T-cell activity (31); and (3) enhancing angiogenesis through VEGF-mediated pathways (32). Recent studies (33, 34) corroborate these mechanisms, linking elevated CA199 to metastatic niche formation and immune tolerance in gastric cancer. Our model advances this paradigm by demonstrating their incremental predictive value when synergized with anatomical staging—a critical improvement over single-modality biomarker studies. Our model advances this paradigm by demonstrating their incremental predictive value when synergized with anatomical staging—a critical improvement over single-modality biomarker studies such as Yang et al.’s peritoneal metastasis nomogram (AUC = 0.75) (23).

The incorporation of pN stage reinforces the mechanistic interplay between lymphatic invasion and TD development. This dual-axis stratification mirrors the biological continuum (4, 5, 17), where advancing N-stage reflects pre-metastatic niche formation (4, 5, 17, 19). Such pathophysiological coherence enhances our model’s translational validity compared to purely statistical prediction tools, bridging histopathological features with metastatic biology.

To ensure generalizability, we further validated the nomogram across key subgroups. In stratified analyses, the model retained high discrimination regardless of tumor location (Antrum: AUC = 0.798; Cardia: AUC = 0.776) or Lauren classification (Diffuse type: AUC = 0.812; Intestinal type: AUC = 0.785) (Supplementary Table 2). These findings align with Gu et al. (14), who emphasized uniform TD prognostic value across anatomical subsites, and Liang et al. (13), who identified Lauren classification as a modifier of TD-associated outcomes. The consistency across subgroups underscores the model’s adaptability to heterogeneous gastric cancer biology.

Methodologically, LASSO regression resolved multicollinearity between tumor stage and biomarkers while preserving clinical interpretability—a key limitation in conventional TD research (35–38). The resultant nomogram outperforms previous single-center models and rivals multicenter algorithms like Li et al.’s dMMR prognostic model (AUC = 0.79) (2), achieving broader applicability through biomarker-driven stratification. These advancements position our tool as a pragmatic solution for preoperative decision-making.

Three limitations merit consideration. First, while bootstrap validation mitigates single-center bias, external verification in ethnically diverse cohorts remains imperative. To address this, we propose a multi-center validation study across three tertiary hospitals in China, with standardized protocols for biomarker measurement and pathological review. Second, exclusion of neoadjuvant therapy recipients may underrepresent aggressive subtypes responsive to systemic therapy, a limitation shared by Fujikawa et al.’s cohort (17), potentially narrowing applicability to treatment-naïve populations. Third, while external validation is essential for clinical adoption, the absence of serum biomarkers (CEA/CA199) in large public databases like SEER currently precludes full validation of our model. To address this, we will validate the pathological components (tumor size, pN stage) using SEER data and collaborate with multi-institutional cohorts to compile biomarker-enriched datasets for comprehensive verification. Future studies integrating molecular data—such as tumor mutational burden, epigenetic alterations, or transcriptomic signatures—could further refine predictive accuracy by elucidating genotype-phenotype correlations. For instance, incorporating liquid biopsy markers (e.g., ctDNA) may capture dynamic metastatic potential, while spatial transcriptomics could map microenvironmental drivers of TD formation at single-cell resolution. Such multi-omics integration would not only enhance risk stratification but also identify actionable targets for precision therapies.

## Conclusion

The proposed TD risk stratification model integrates clinicopathological variables and serum biomarkers into a visualized prognostic framework, demonstrating high predictive accuracy with robust validation in both cohorts. Its biological plausibility (via CA199-driven mechanisms) and consistent performance across tumor subtypes reinforce clinical utility in diverse populations. This computational tool bridges a significant unmet need in preoperative risk stratification for locoregional gastric cancer (Stage I-III), offering clinically actionable insights to guide individualized surveillance intervals and adjuvant treatment allocation decisions. Upon successful multicenter external validation, this model holds potential to streamline evidence-based TD management protocols while establishing a scalable methodology for incorporating novel molecular signatures into future iterations.

## Data Availability

The raw data supporting the conclusions of this article will be made available by the authors, without undue reservation.
